# Supplementary material for: Sexual assault and the matrix of harm: Sexual assault survivors narrate their whole lives in more negative ways
Source: PLoS One. 2024 Jun 12;19(6):e0297650. doi: 10.1371/journal.pone.0297650 (PMC11168687; doi:10.1371/journal.pone.0297650)
Supplement: Attachment — Submitted filename: PLOS REVISION MEMO.docx [file pone.0297650.s001.docx]

Shamus Khan

Willard Thorp Professor

Sociology & American Studies

Princeton University

**RE:** Revision and Resubmission to PLOS ONE

**MANUSCRIPT ID:** PONE-D-23-23689

**MANUSCRIPT TITLE:** Sexual Assault and the Matrix of Harm: How Sexual Assault Survivors narrative their whole lives in more negative ways

October 30, 2023

Dear Dr. Shrestha -

We wish to thank you and the two reviewers for their generous and helpful feedback on our manuscript. We are particularly appreciative of your guidance in this process, and your work to advance our manuscript through the review process. From experience we know how challenging it can be to find expert reviewers and we appreciate your work to do so. We have significantly edited the manuscript considering the reviews and added a new section requested by you and reviewer 1.

In reading the reviews we suspect we have a different disciplinary and epistemic understanding to Reviewer 1. This is to be expected for a generalist journal like PLOS ONE, and we believe we have edited our manuscript in ways that preserve our understanding of the scientific enterprise while respecting the different approach represented by Reviewer 1.

We have grounded our work in a conversation with other related work and credited the authors of that work for inspiring us. As we note in the paper, Beth Richie writes on a “matrix of violence” that Black women experience, from the state, markets, and in domestic life. We use her approach to outline what we call a “matrix of harm” that survivors of sexual assault (including men and people who are not Black) experience. We seek to provide extensive empirical evaluations of such a matrix. This means not simply providing one finding, but critically evaluating and testing our finding, and consistently asking, “how might we be wrong?” This results in our undertaking a series of evaluations of our data to see its limits and weakness; the core of our epistemic approach is based on the assumption that science advances through negation. We understand this can seem excessive if the answer seems obvious, but from our perspective it is one of the few ways we can feel secure in our knowledge.

We have added some clarity to the paper out of respect for Reviewer 1’s reading. The main finding in this paper is novel to the field: people who have experienced sexual violence, some of which happened decades ago, described their lives in ways that are filled with more anger, disgust, fear, and sadness, and less anticipation, joy, and trust. This finding is generated using a random population dataset (something that is relatively novel for the field) and using a range of methodologies, including sentiment analysis (again, novel to the field). This finding is particularly surprising as it is observable before an interview subject is even asked about experiences of assault. It also has important implications beyond sexual violence research. We contextualize this finding with other potential sources of harm in people’s lives to help interpret the magnitude of the effect.

We appreciate your suggestion, and the suggestion of reviewer 1 to be clearer about these contributions. We have followed all recommendations for our revision and added a section of the paper that does this. That section, entitled, “Contributions” is longer than the single paragraph that Reviewer 1 asked for. We also combined it with our previous conclusion section. As an appendix to this response letter, we list every request or suggestion, and outline the action we have taken to address the concern. In almost every instance we made significant edits to address the issues raised within this review process.

We look forward to your evaluation of this manuscript, which we believe has been improved by your guidance and that of our reviewers.

Best,

Shamus Khan

Professor of Sociology & American Studies

Princeton University

[shamuskhan@princeton.edu](mailto:shamuskhan@princeton.edu)

(917) 428-3492

| **Person** | **Comment** | **Section** | **Action** |
| --- | --- | --- | --- |
| Journal Policy | Ensure that your manuscript meets PLOS ONE's style requirements, including those for file naming. | Entire paper | We have hired a copyeditor to make sure our paper is professionally edited and conforms to all guidelines. |
| Journal Policy | In your Data Availability statement, you have not specified where the minimal data set underlying the results described in your manuscript can be found. PLOS defines a study's minimal data set as the underlying data used to reach the conclusions drawn in the manuscript and any additional data required to replicate the reported study findings in their entirety. All PLOS journals require that the minimal data set be made fully available. If there are ethical or legal restrictions to sharing your data publicly, please explain these restrictions in detail. | Main Body | A new section to the paper has been added, which includes information both about data availability and ethics (see below). Interviews from the American Voices Project considered many sensitive topics, including but not limited to experiences of sexual assault. Given the breadth and detail of the interview protocol, subjects are at extreme risk of being identifiable. This limits the broad public availability of data. Data from this project is available to researchers upon application through the American Voices Project, based at Stanford University. Interested scholars may be granted access to data upon successful application to their own institutional review board, and, their following protocols outlined by the American Voices Project. Interview subjects, as part of the IRB process, were promised that, “All members of our research team are legally required to keep all information provided by participants confidential and secure. We will never identify you as having participated in the study.” This means we cannot provide data; however scholars interested in evaluating our results may apply for access (which is free, and a relatively easy process). |
| Journal Policy | Please ensure that you have an ORCID iD and that it is validated in Editorial Manager | Title Page | ORCID ID added to editorial manager |
| Journal Policy | Please ensure that you include a title page within your main document. You should list all authors and all affiliations as per our author instructions and clearly indicate the corresponding author. | Title Page | Title page has been added with all author affiliations and with clear indication of corresponding author. |
| Journal Policy | Please include your full ethics statement in the ‘Methods’ section of your manuscript file. In your statement, please include the full name of the IRB or ethics committee who approved or waived your study, as well as whether or not you obtained informed written or verbal consent. | Methods | A new section has been added that includes both the ethical approval information from the IRB as requested, as well as information on data availability (see above). |
| Journal Policy | Please include a copy of Table 4 and 6 which you refer to in your text on page 20 and 22. | Findings | Table 4 has been removed from the appendix and placed into the main text. Table 6 has been added to the main text of the paper. |
| Journal Policy | Please review your reference list to ensure that it is complete and correct. If you have cited papers that have been retracted, please include the rationale for doing so in the manuscript text, or remove these references and replace them with relevant current references. Any changes to the reference list should be mentioned in the rebuttal letter that accompanies your revised manuscript. If you need to cite a retracted article, indicate the article’s retracted status in the References list and also include a citation and full reference for the retraction notice. | Cites | We have hired a copyeditor to make sure our paper is professionally edited, and conforms to all guidelines.  We do not reference any retracted papers. |
| Editor | Please include a minor revision that includes a statement on “What this study adds to our current knowledge” | New Section | A new section has been added as requested. It is entitled, “Contributions.” This section is after our limitations section and has been combined with our previous section entitled “conclusion.” It draws upon our previous conclusion but adds considerably more material as well as clarifying language about what the novel contributions of this paper are. |
| Reviewer 1 | The author of this paper, after using scientific instruments such as “using quantitative sentiment analysis”, arrives at the conclusion, that those who have experienced sexual assault narrate their lives more negatively than those who did not. Furthermore, the author says himself in his abstract, “Overall, this paper suggests that sexual violence is part of what we call, following Beth Richie, a “matrix of harm” that structures people’s lives.” Thus, the author is admitting that people have already said, what he has scientifically “discovered” in this paper. | Abstract intro | No action taken. The reviewer’s comment, “the author is admitting that people have already said, what he has scientifically “discovered” in this paper” seems to misunderstand the role of Richie within our paper. We have added material to make sure other readers do not come away with the same impression about our work. Richie does not develop the idea of a “matrix of harm” and instead introduces the concept of a “matrix of violence.” The types of violence Richie speaks of are multiple and Richie is interested in the specific case of Black women. She is not interested in life narration (our object of inquiry). Our paper builds off Richie, and we felt it important to acknowledge that connection to her pioneering work, which inspired our own ideas. But we address only sexual violence, and we also deal with data on men; most of our respondents are not Black. The concept we use is different from hers, our empirical data draw upon a very different population, and our object (sexual violence) is also different. This may be a difference in disciplinary approach wherein it is customary to credit others whose work inspires us, even if that work deals with different cases, draws on different empirical material, and generates a different concept. |
| Reviewer 1 | The author then goes on for a detailed definition/analysis/discussions of oft-repeated topics like “sexual violence” “matrix of harm”. “Impacts of negative statement” etc. The author also makes use of several interviews carried out under the “American Voices Project” From 2019 to 2021, and then describes a complex “Analytic Strategy” to come to the above conclusion. | Entire paper | No changes requested; no changes made. |
| Reviewer 1 | I think even an ordinary layman knows this already. Even from personal experiences, most of us know that any incident which is violent and brings harm to us, fills our life with negative emotions. Thus, I do not find anything new in this paper. If the author may resubmit his paper with just one additional paragraph entitled – “What this study adds to our current knowledge” it would make everybody understand properly what exactly is the new content in this paper. Importantly, in many journals, this column is an essential requirement. | Entire paper | Again, our approach to the scientific enterprise is likely different from this reviewer. The findings themselves may not be surprising (though as experts in this field, they were to us!). We see an important difference between having an impression about something and rigorously and systematically generating empirical observations to establish an impression as a scientific finding. There are many things the layperson “knows” about sexual violence that do not have a strong empirical foundation. And there are many things the lay person “knows” that are demonstrably false. From our perspective, knowledge progresses by incremental findings as well as the continued evaluation of what previous scholars have found. This research developed many new findings; a new section has been added, as requested by the reviewer. That section is entitled “Contributions.” We hope this addresses their concerns and helps the paper in having other readers not have the same reaction. |
| Reviewer 1 | I am just wondering if this might be a case of “complexity bias”, where individuals have a tendency to overcomplicate simple things. But I will give the author a chance, and repeat my simple request. Add a final note – What exactly does this study add to our knowledge, and how does that “additional knowledge” help us in any manner. | Entire paper | We do undertake a range of empirical tests of our findings. The purpose of these is to try to establish, as rigorously as possible, the findings themselves. Instead of accepting a single test of a finding because it conforms to what we would expect, our approach is to be skeptical and to try and negate the findings. Our inability to do so gives greater strength to the position that we have a finding that is empirically grounded. A new section, “Contributions,” has been added, as requested by the reviewer. We hope this addresses their concerns and helps the paper in having other readers not have the same reaction. |
| Reviewer 2 | The submitted manuscript hold its value in the field of forensic medicine. Very good presentation of data, results and other relevant facts. Limitations are also defined in the manuscript appropriately. Manuscript is worth publishing. | Entire paper | The authors thank the reviewer for their generous read of our paper. |
